# Impact of COVID-19 on antibiotic usage in primary care: a retrospective analysis

**DOI:** 10.1038/s41598-024-55540-5

**Published:** 2024-02-27

**Authors:** Anna Romaszko-Wojtowicz, K. Tokarczyk-Malesa, Anna Doboszyńska, K. Glińska-Lewczuk

**Affiliations:** 1 Department of Pulmonology, School of Public Health, Collegium Medicum, University of Warmia and Mazury in Olsztyn, Jagiellońska 78, 10-357 Olsztyn, Poland; 2 Department of Family Medicine and Infectious Diseases, Collegium Medicum, School of Medicine, University of Warmia and Mazury in Olsztyn, Olsztyn, Poland; 3 Department of Water Resources, Climatology and Environmental Management, University of Warmia and Mazury in Olsztyn, Olsztyn, Poland

**Keywords:** Biostatistics, Respiratory tract diseases, Antimicrobial therapy

## Abstract

The COVID-19 pandemic has contributed to many changes in the medical practice, including a wider access to tele-consultations. It not only influenced the type of treatment but also shed light on mistakes often made by doctors, such as the abuse of antibiotics. This study aimed to evaluate the antibiotic treatment, and the impact of the COVID-19 pandemic on antibiotic prescribing during a GP’s visit. The retrospective medical history analysis involved data from a first-contact medical center (Pantamed, Olsztyn, Poland), from 1 January 2018 to 31 May 2023. Quantities of prescribed antibiotics were assessed and converted into the so-called active list for a given working day of adult patients (> 18 years of age). Statistical analysis based on collective data was performed. During the COVID-19 pandemic, a decline in the number of medical consultations has been observed, both remotely via tele-medicine and in personal appointments, compared to the data from before the pandemic: n = 95,251 versus n = 79,619. Also, during the COVID-19 pandemic, there was a decrease in the total amount of prescribed antibiotics relative to the data before the pandemic (2.44 vs. 4.54; *p* > 0.001). The decrease in the quantities of prescribed antibiotics did not depend on the way doctor consultations were provided. The COVID-19 pandemic has contributed to changing the family doctors’ management of respiratory infections. The ability to identify the etiological agent—the SARS-COV2 virus—contributed to the reduction of the antibiotics use.

## Introduction

On 11 March 2020, WHO announced the outbreak of a pandemic caused by a coronavirus (COVID-19), a disease caused by the severe acute respiratory syndrome virus 2 (SARS-CoV-2). This disease can be asymptomatic, with only mild symptoms, mainly from the respiratory tract, or can lead to acute respiratory insufficiency. In uncomplicated cases, as an isolated viral infection, it requires the treatment of symptoms, which is a contraindication to antibiotics use. The widespread availability of tests to detect the SARS-CoV-2 virus enabled simple and rapid diagnosis of this illness, which should also help to reduce the administration of antibiotics. Overuse of antibiotics leads to various complications, with microbiological resistance being one of the most significant^1^. According to the CDC (Centers for Disease Control and Prevention), more than 2.8 million antibiotic-resistant infections occur in the United States each year, and more than 35,000 people die as a result^2^. It is estimated that the global consumption of antibiotics rose by circa 65% between 2000 and 2015^3^. The increase in antibiotic consumption between 2019 and 2020 in the USA resulted in a 15% rise in antibiotic resistance, contributing to a higher number of deaths, primarily due to nosocomial infections^1^.

With a similar range of symptoms, viral infections of the upper respiratory tract are conducive to a belief that a common cold or influenza is an indication for antibiotic treatment^4–8^. Most patients do not understand the notion of antibiotic resistance. According to Brookes-Howell et al., as many as 35% of patients attribute the resistance to antibiotics to themselves (‘this antibiotic doesn’t work for me’)^9^. It is estimated that over 30% of patients in Poland occasionally resort to self-medication with antibiotics, which confronts doctors with a very difficult decision (no possibility of making a retrograde assessment) to discontinue treatment^10^. Consumption of antibiotics is seasonal, increasing in winter months and decreasing in early spring and summer, which is associated with a rise in influenza-like infections^11–13^.

The purpose of this study has been to evaluate the use of antibiotics in the first-contact doctor’s office, and to assess the impact of the COVID-19 pandemic on prescribing antibiotics.

## Material and method

In order to reliably verify the recommended antibiotic therapy, data from the National Health Fund (NFZ), which is the healthcare payer in Poland, were used. The doctor-payer communication channel is used to transmit information about e-prescriptions issued. Each information package of this kind contains both the EAN code of a prescription and its international classification called ATC (Anatomical Therapeutic Chemical Classification System). This classification allows one to identify groups of drugs, and not just to see their trade or chemical names. For example, macrolides are coded as J01FA^15^. This information is encoded in every medical prescription and is therefore available for analysis, which was taken advantage of in our database.

The most recent data (on 2 April 2023) issued by the Ministry of Health, in Poland, indicate that 35 924 946 tests for COVID-19 had been used^14^. Thus, the COVID-19 pandemic seems to have been an ideal period (a natural experiment) to verify the therapeutic choices of doctors regarding antibiotics and antibiotic-based treatment.

### Data collection methodology

A family doctor in Poland works on the so-called active list (people who have chosen a doctor/clinic). This list is quite stable, but in a practice with several thousand people it changes by about 100 people a month (enrolled, discharged, deceased). In addition to these admissions, the family doctor is obliged to provide assistance to people who seek medical help but and live in another part of the country (e.g. people looking for medical consultation during holidays). The program built a database consisting of the active list and omitting ad hoc visits. The prerequisite was that the patient in a given month was under the care of the clinic for the entire month. People who signed up or resigned due to other reasons during a given month were not included in that month. The program generated a database for each subsequent month from 1 January 2018 to 31 December 2022. Then, these databases were merged. The maximum date of birth was also determined for each month (so as not to include persons under 18 years of age). By building monthly databases, the program added the occurrence of events specified in the search to the patient (if any). To avoid double coding of the same diseases, arbitrary minimum time intervals were adopted (Table Z—supplementary files). In Poland, where the national healthcare is free for the patient, it happens quite often that a patient diagnosed with a viral infection visits his family doctor 2–3 times in a short period of time (for example, on Monday he is examined but refuses to take a sick leave, on Tuesday he returns to claim his sick leave and on Wednesday he makes another appointment because he is convinced that he will not be cured without antibiotics). To account for such patterns, the assumed intervals were 30 days for COVID-19, 7 days for other viral infections, 14 days for illnesses suggesting a bacterial background, and a 10-day interval for antibiotics (for details, see Table Z—supplementary files). The program treated repeated events within a time shorter than the specified minimum interval as one. A case was included in the analysis if the product of events was met, thus urinary tract infections for example were excluded from the analysis (no ICD-10 code searched, ATC code searched).

The retrospective cross-sectional analysis included medical data of adults, i.e. aged 18 and older (on the date of the visit), who visited a doctor, either in the form of a remote consultation (tele-admission understood as tele-consultation) or as an on-site visit, from 1 January 2018 to 31 May 2023. In that period, the medical clinic Pantamed, Olsztyn, Poland, served an active list of patients aged over 18 years from 11 341 to 12 615 persons. In total, 716 242 records with data from medical consultations were submitted to the analysis.

All collected data were divided into 3 groups: before the COVID-19 pandemic (1 January 2018–31 May 2020), during the pandemic (1 June 2020–30 June 2022), and after the COVID-19 pandemic (1 July 2022–31 May 2023). The selection of the date of the actual (not official) termination of the pandemic (or its influence on the operation of first-contact medical centers) was based on the analysis of a biplot of discriminant, which confirmed that 93.9% of data were classified correctly.

### Statistical analyses

To determine the level of antibiotic use in three periods over 2019–2023, namely: before, during, and after the COVID-19 pandemic, the index of average antibiotic consumption was related to a daily intake by a patient aged 18 years. To statistically evaluate the significance of differences in consumption of seven antibiotic groups, such as β-lactams (J01C), amoxicillin + clavulonian acid(J01CR), macrolides (J01F), azithromycin (J01FA10), tetracyclines (J01A), and quinolones (J01M), in the three periods, one-way analysis of variance followed the test for normality distribution of variables (Kolmogorov–Smirnov test, p < 0.05).

In the procedure to detect variations in antibiotic consumption, a univariate ANOVA was employed to determine if there were statistically significant differences between the means of each group of antibiotics in three COVID-related periods. The differences were analyzed with a multiple comparison test (HSD Tukey's test, df = 62) as a *post-hoc* procedure.

Afterwards, linear discriminant analysis (DA) was implemented to determine if the time data were properly classified to the COVID-related periods (before, during, and post-COVID-19 pandemic). The use of DA enabled us to verify the accuracy of the a priori classification of COVID-related groups: before, during, and after the pandemic. DA was performed based on both frequencies of patient tele- and on-site admissions as well as the use of 6 antibiotics.

Statistical analyses were performed using Statistica™ 13.1 (TIBCO Software Inc. Palo Alto, US, 2021) and Past 4.03^16^.

### Ethics

The study presented in this paper is a retrospective analysis of patients' medical records, in which no personal data of the patients were used or processed. Because the study was retrospective, written informed consent could not be received from all patients.

An ethics approval from the Bioethics Committee at the Warmia and Masuria Medical Chamber in Olsztyn (Resolution No. 30/2023/VIII) was obtained, and informed consent was waived by the approving ethics committee. All methods were performed in accordance with relevant guidelines and regulations.

## Results

In this study, patients ≥ 18 years old comprised a group of 87.3% of the whole population considered. The share of patients ≥ 18 during the pre-Covid period amounted to 88.0%, 89.2% during the Covid pandemic, and 82.6% in the post-Covid period (Table 1). Before the COVID-19 pandemic, patients at least 18 years old were given 1,183 medical consultations online, which corresponded to 1.2% of all consultations, and 94,068 on-site consultations (98.8%). During the pandemic, there were 44,454 (55.8%) and 35,165 (44.2%) online and on-site consultations, respectively, and after the pandemic, the respective figures were 5,544 (23.8%) and 35,069 (76.2%).

**Table 1:** Number of admissions of all patients and patients aged 18 + before, during, and after COVID-19.

Period	Patients 18 +				% of > 18 + in total	All patients			
	Avr	sum	% sum	% admissions in total*		avr	sum	% sum	% in total*
Tele-admissions									
Pre—COVID 19	44	1183	2.3	1.2	94.2	47	1256	2.2	1.2
COVID 19	1852	44,454	86.9	55.8	92.1	2011	48,271	86.3	54.1
Post—COVID 19	396	5544	10.8	13.7	86.8	456	6389	11.4	13.0
Total	787	51,181	100	23.8	91.5	860	55,916	100	22.7
On-site admissions									
Pre—COVID 19	3484	94,068	57.3	98.8	87.9	3963	107,013	56.1	98.8
COVID 19	1465	35,165	21.4	44.2	85.7	1709	41,025	21.5	45.9
Post—COVID 19	2505	35,069	21.3	86.3	81.9	3058	42,805	22.4	87.0
Total	2528	164,302	100	76.2	86.1	2936	190,843	100	77.3
Tele- and on-site admissions									
Pre—COVID 19	3528	95,251	44.2	100	88.0	4010	108,269	43.9	100
COVID 19	3317	79,619	36.9	100	89.2	3721	89,296	36.2	100
Post—COVID 19	2901	40,613	18.8	100	82.6	3514	49,194	19.9	100
Total	3315	215,483	100	100	87.3	3796	246,759	100	100

*% admissions in total” refers to calculating the % share of tele-admissions or on-site admissions during a pre-COVID, during COVID, or post-COVID time relative to the sum of such admissions in the same period.

**Figure 1:**
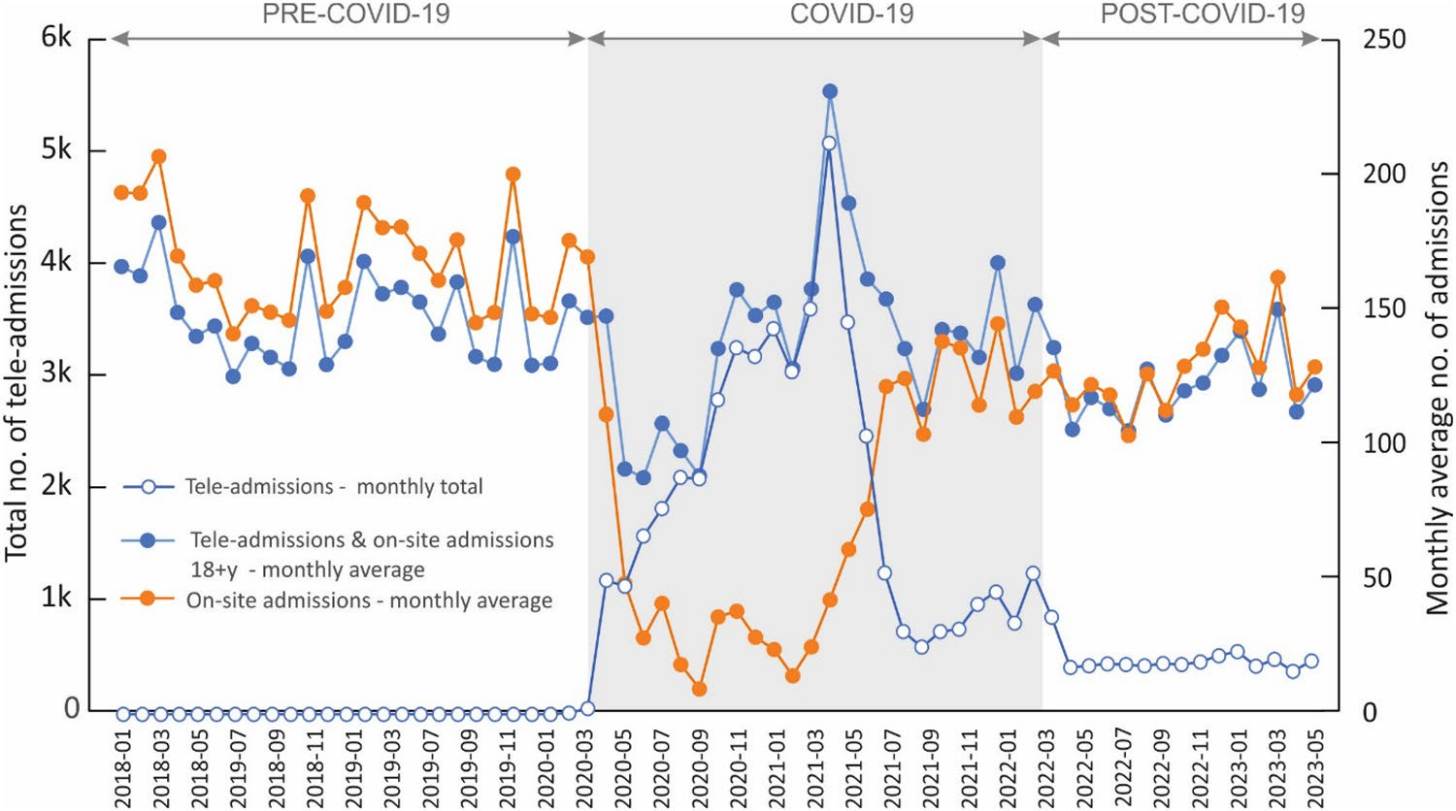
Changes in the number of admissions of patients aged 18 + years in the period before, during, and after the COVID-19 pandemic, depending on the type of admissions: on-site and tele-admissions, as well as the total number of admissions.

Figure 1 shows data concerning the number of admissions in each of the analyzed months, divided into the types of visits: on-site and tele-medicine, and the total number of consultations.

**Figure 2:**
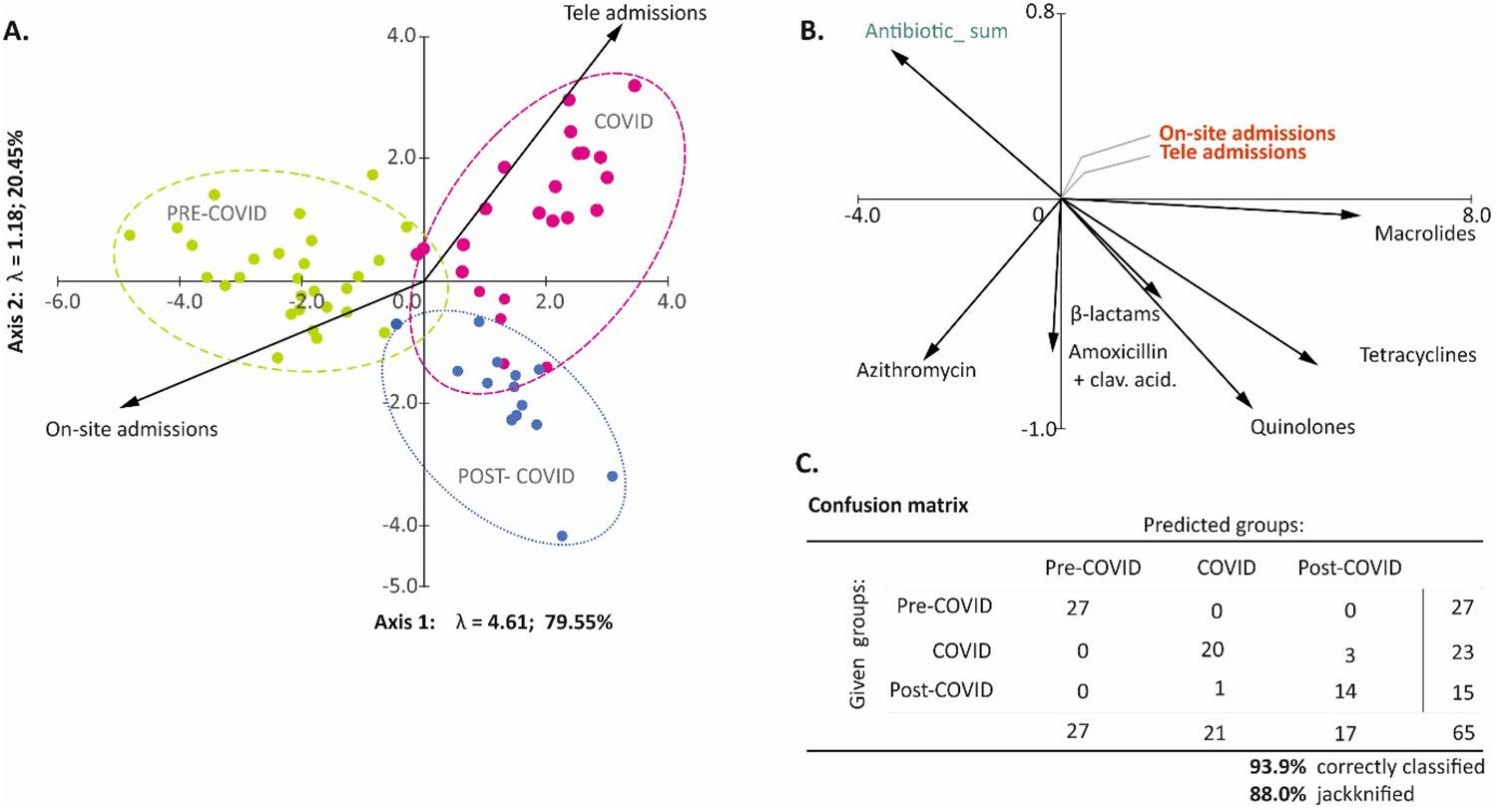
(**A**) Biplot of discriminant analysis (DA) showing the classification of monthly rates of antibiotic use in pre-COVID, during COVID, and post-COVID periods. (**B**) Summary of classification given and predicted temporal groups with jackknife cross-validation. (**C**) Confusion matrix for given and predicted pre-COVID, COVID, and post-COVID groups with quality of the classification.

Discriminant analysis (DA), a multivariate statistical technique, allowed the classification of the antibiotic consumption rate (per person per month), and provided the best separation of the temporal groups previously (a priori) defined: before, during, and after COVID-19 (Fig. 2a). Based on the 65 sets of monthly data concerning the use of 6 antibiotics and the type of consultation with patients, DA (Fig. 2b) showed that the three predefined groups were correctly assigned 93.9% of the time and 88% when cross-validated by the jackknife principle.

A significant decrease in the number of visits (both on-site and tele-consultations) and the number of consultations provided in the doctor's office was evident during the COVID-19 pandemic During that period, a new form of medical service emerged, namely tele-admission, which remains available after the pandemic. However, the number of tele-consultations since May 2020 has remained low, accounting for only about 11% of the total number of admissions.

Due to fluctuations in the number of patients registered with the clinic, which can result from factors such as deaths or transfers to other clinics providing basic medical care, the authors utilized an indicator. This indicator is calculated as the quotient of the number of events (visits to the clinic) divided by the total number of registered patients on a given day, multiplied by the number of working days in a given month. Based on this indicator, Table 2 presents data regarding the use of antibiotics, categorized into three periods: before, during, and after the COVID-19 pandemic.

**Table 2:** Comparison of the indicator of the use of antibiotics per patient/working day in groups according to the time intervals before, during, and after the COVID-19 pandemic. The differences between the means of groups were tested with multiple comparison tests (HSD Tukey’s test, df = 62) as a *post-hoc* procedure of one-way ANOVA.

Antibiotic	PRE-COVID	COVID	POST-COVID	*p*
Any antibiotic (J01)	4.54^b^	2.44^a^	2.84^ab^	> 0.001
β-lactams (J01C)	1.56^b^	0.72^ab^	1.01^ab^	> 0.001
β-lactams (J01CR02)	0.77^b^	0.39^a^	0.48^ab^	> 0.001
macrolides (J01FA)	1.49^b^	0.89^a^	0.95^ab^	> 0.01
azithromycin (J01FA10)	1.14^b^	0.74^a^	0.74^a^	> 0.05
tetracyclines (J01AA)	0.18^b^	0.09^a^	0.07^a^	> 0.01
quinolines (J01GA)	0.44^b^	0.26^a^	0.29^ab^	> 0.001

Data with different superscript letters denote significant differences.

**Figure 3:**
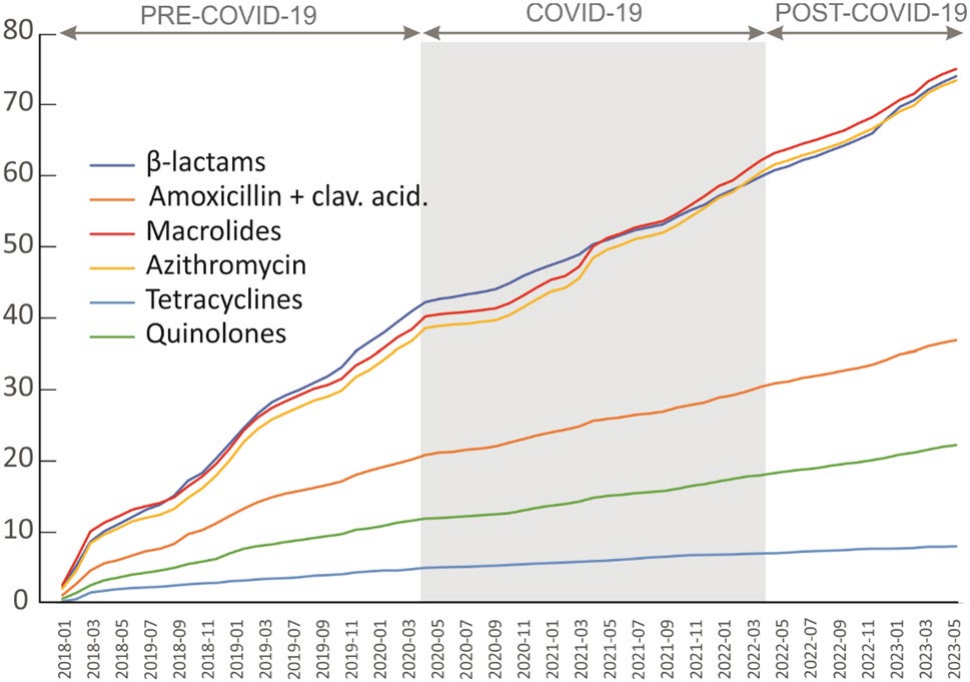
Cumulative curves of antibiotics use (Pantamed) related to the number of patients. The grey area denotes the COVID-19 pandemic period.

Figure 3 presents cumulative data on antibiotic usage. The diagram illustrates a notable reduction in antibiotic prescriptions, particularly noticeable for all β-lactams and macrolides, including azithromycin, during the pandemic, particularly in its first year.

## Discussion

The COVID-19 pandemic had a significant impact on the entire healthcare sector. For epidemiological reasons, tele-consultations, which were previously rarely used in the state healthcare system, became essential to maintain the continuity of medical services.. As shown in Fig. 1, there was a substantial increase in remote healthcare services in March 2020, coinciding with a decrease in patients' visits to GP's offices. As the pandemic escalated, the primary focus shifted to the care of COVID-19 patients, leading to a significant reduction in face-to-face consultations with doctors, primarily limited to emergency cases, in order to minimize the risk of disease transmission between doctors and patients^17^. Between June and September 2021, this trend began to reverse, with a decrease in tele-admissions and a return to on-site admissions in clinics. At the same time, according to data issued by the Ministry of Health in Poland, the pandemic came to a halt, and the number of new cases was in the range of 100–200 new cases/day^18^.

In December 2020, COVID-19 vaccines became available in Poland, and the highest vaccination coverage of the population was achieved by the spring of 2021, contributing to a renewed increase in the number of on-site medical consultations^19^.

COVID-19 is a viral infection caused by the SARS-CoV2 virus, which can be treated with such antiviral medications as nirmatrelvir, ritonavir, remdesivir, and molnupiravir^20–23^. According to the data presented in this article, there was a significant reduction in the administration of antibiotics from January to April 2020. However, in October 2020, the use of antibiotics began to gradually increase. At the onset of the COVID-19 pandemic, there was a decrease in the overall use of antibiotics (Fig. 3).

The results presented in Table 1 indicate a noticeable difference in antibiotic use before, during, and after the COVID-19 pandemic. Relative to the period preceding the pandemic, the number of prescribed antibiotics during the pandemic decreased, both in total and divided into groups of antibiotics. This trend is observed for each analyzed group of antibiotics.However, there is a discernable difference in the use of antibiotics before and after the COVID-19 pandemic, applying to each prescribed antibiotic.

The gradual increase in the quantities of prescribed antibiotics observed later during the COVID-19 pandemic may be attributed to subsequent waves of the pandemic and secondary bacterial complications.. Shafran et al., based on data collected from hospitalized patients, demonstrated that patients with COVID-19 had more documented secondary bacterial infections than patients with influenza, and these infections were not correlated with the death of patients with COVID-19, but not in patients with influenza^24^. On the other hand, Nandi et al. demonstrated that the bacterial co-infection index for patients with COVID-19 was lower than 10%, yet antibiotics were prescribed to over 75% of COVID-19 patients^25^. This approach is linked to the development of antimicrobial resistance. The CDC estimates that from 30 to 50% of all antibiotics prescribed in ambulatory care are unnecessary^2^.

An article published in Lancet in February 2020 contained an analysis of monthly data from the company IQUVIA MIDAS regarding sales volumes of four groups of broad-spectrum antibiotics: cephalosporins, penicillin, macrolides, and tetracyclines (ATC classification was employed)^25,26^. This article, based on data from 71 countries covering the period from January 2018 to May 2020, showed that the sales of all four groups of antibiotics decreased rapidly in April and May 2020. Since May 2020, there has been a gradual increase up to the level similar to that before the pandemic. Another high-impact journal article, authored by Allard et al. in 2023, showed that antibiotic use increased during the COVID-19 pandemic; however, this study focused only on data from hospitalized patients^3^. The apparent contradiction between these two studies can be explained by our results, based entirely on data from an outpatient clinic. It is worth noting that the clinic returned to its normal work in the final stage of the pandemic. Arbitrarily assuming its end date (May 5, 2023, according to WHO) would be an error in our analysis^27^. Our data (Fig. 2) suggest that the actual end of the direct impact of the pandemic on the healthcare system in our country occurred in March/April 2022. Figure 2, which demonstrates that 93.9% of cases were correctly classified according to the time intervals assumed in our study, supports our assumption.

Our study has a few limitations. First of all, it is a retrospective study conducted in one healthcare center, and its results depend on the accuracy of the center's medical records and diagnosis coding. The study may also be affected by selection bias, as the decision to prescribe antibiotics was made by individual doctors based on their subjective assessment of the severity of each case and the overall clinical picture of the patient. In a retrospective assessment, as explained above, it is impossible to question the reasons for these earlier decisions. Secondly, considering the local screening policy, not every admitted patient was tested for COVID-19, and there is a possibility that some COVID-19 test results may have been falsified by patients during tele-admission visits to avoid isolation. However, the picture of antibiotic use emerging from the data presented in this article is difficult to challenge. In our opinion, widespread access to screening tests such as COVID-19 tests, and consequently, the confirmation of viral infections, had a positive impact on reducing antibiotic administration in outpatient healthcare.

## Conclusions

The COVID-19 pandemic is a lesson for doctors in first-contact health clinics, showing that the reduction in the administration of antibiotics is possible and recommended. The amounts of antibiotics administered in outpatient health care decreased during the COVID-19 pandemic, and despite their slight increase later on, their prescribing remains lower than before the pandemic (Fig. 3). Our observations suggest that future research should carefully consider the timing of the pandemic, including its end date. Using an arbitrarily chosen end date, such as May 5, 2023, can potentially introduce inaccuracies in study findings.

### Supplementary Information

Supplementary Tables.

## Data Availability

The data that support the findings of this study are available on request from the corresponding author.
