# Supplementary material for: Impact of COVID-19 on antibiotic usage in primary care: a retrospective analysis
Source: Sci Rep. 2024 Feb 27;14:4798. doi: 10.1038/s41598-024-55540-5 (PMC10899221; doi:10.1038/s41598-024-55540-5)
Supplement: Supplementary file 1 — Supplementary Tables. [file 41598_2024_55540_MOESM1_ESM.docx]

Table Z. Methodology of building the database, in the search content in lines 1-3 ICD-10 codes were used, in the remaining ATC codes, delta - minimum time interval.

| **Reason** | **Delta [days]** | **Search content */**** |
| --- | --- | --- |
| COVID-19 | 30 | U07.1 U07.2 |
| Viral infection | 7 | J00 J04 J05 J10 J11 J12 |
| Bacterial infection | 14 | J01 J02 J03 J06 J13 J14 J15 J16 J17 J18 J21 |
| Any antibiotic | 10 | J01 |
| Betalactam antibiotic | 10 | J01C |
| Amoxicillin with clavulanic acid | 10 | J01CR |
| Makrolides | 10 | J01F |
| Azithromycin | 10 | J01FA10 |
| Tetracyclines | 10 | J01A |
| Quinolones | 10 | J01M |

* ICD 10 code; ** ATC classifier
